# Diagnosis of prostate cancer in primary care: navigating updated clinical guidance

**DOI:** 10.3399/bjgp23X731769

**Published:** 2023-01-27

**Authors:** Samuel WD Merriel, Andrew Seggie, Hashim Ahmed

**Affiliations:** Centre for Primary Care and Health Services Research, University of Manchester, Manchester.; Prostate Cancer UK, London.; Department of Surgery and Cancer, Imperial College London; Chair of Urology, Imperial College Healthcare NHS Trust, London.

## PROSTATE CANCER IN THE UK

Prostate cancer became the most common cancer diagnosed in males in the UK in 2018, with around 52 300 new cases.^1^ The COVID-19 pandemic impacted prostate cancer diagnoses more than any other tumour type and up to 14 000 fewer prostate cancer cases were detected in the first 2 years of the pandemic than would be expected based on long-term trends.^2^^,^^3^ This was thought to be in part due to fewer patients coming forward to their GP with symptoms warranting an urgent suspected cancer (‘2-week wait’) referral or to discuss opportunistic prostate-specific antigen (PSA) screening. Early-stage diagnosis (stage I/II) of clinically significant prostate cancer is crucial for improving outcomes for patients with prostate cancer. Five-year survival for patients with prostate cancer diagnosed at stage I or II is close to 100%, whereas for patients diagnosed at stage IV, around 50 out of every 100 men — around 50% — will survive their cancer for 5 years or more after they are diagnosed.^4^ The delays in prostate cancer diagnosis for thousands of patients as a result of the pandemic could have significant long-term effects. This will make achieving the NHS Long Term Plan aim of diagnosing 75% of patients with early-stage cancer by 2028 all the more difficult.^5^

The vast majority of patients with prostate cancer are diagnosed following a referral from their GP. Over half of these patients are referred via the 2-week wait pathway, and another quarter following a routine GP referral to urology.^6^ Prostate cancer diagnoses following emergency presentation are an uncommon occurrence.^6^ Prostate cancer is usually suspected in primary care with either an abnormal digital rectal examination (DRE) of the prostate or an elevated PSA level. The presence of one of these clinical features is an indication for a 2-week wait referral according to National Institute for Health and Care Excellence (NICE) guidance 12 (NG12).^7^

## CURRENT CLINICAL GUIDANCE

NICE guidance related to prostate cancer diagnosis and management (NG12 and NG131) underwent an evidence review and update in late 2021. The 2015 version of NG12 recommended GPs perform a DRE and PSA for patients presenting with symptoms associated with prostate cancer, such as lower urinary tract symptoms. NG12 advocated using age-adjusted PSA ranges to identify males at higher risk of prostate cancer for 2-week wait referral but did not actually specify what ranges of PSA should be considered abnormal for different age groups. This led to significant variation in care for patients with suspected prostate cancer in England.^8^ The updated 2021 version of NG12 continues to advise the use of age-adjusted PSA ranges and specified what these levels should be (see Table 1). These recommendations are based on indirect evidence from PSA screening trials as there are no published studies of the diagnostic accuracy of PSA in a primary care setting to date.

**Table 1.** Age-specific PSA thresholds for people with possible symptoms of prostate cancer^6^

**Age, years**	**PSA threshold (μg/L)**
<40	Use clinical judgement
40–49	>2.5
50–59	>3.5
60–69	>4.5
70–79	>6.5
>79	Use clinical judgement

*PSA = prostate-specific antigen.*

NG12 does not make any recommendations with regards to PSA screening for prostate cancer in asymptomatic patients, and regular PSA-based screening is currently not recommended by the UK National Screening Committee. Guidance for GPs in the use of opportunistic PSA screening comes from the Prostate Cancer Risk Management Programme (PCRMP). Guidance from PCRMP was first released in 2009, recommending that any asymptomatic male over the age of 50 years could undergo opportunistic PSA screening following a discussion with their GP about the pros and cons of having a screening PSA test. The initial PCRMP guidance also recommended age-adjusted PSA ranges, but a subsequent update altered this to a PSA threshold of 3 ng/mL regardless of the patient’s age, which was in line with the approach followed in two of the largest PSA screening trials.^9^

For patients being referred with a suspicion of prostate cancer, 72% of areas across the UK are able to offer a pre-biopsy multiparametric MRI (mpMRI) scan.^10^ Pre-biopsy mpMRI scan can help up to a third of males avoid a subsequent biopsy altogether.^11^ More recently, NICE have recommended transperineal biopsy under local anaesthetic (LATP) because the procedure offers reduced biopsy-related infection rates, while maintaining cancer detection rates, compared to traditional transrectal ultrasound guided (TRUS) biopsy.^12^

## DIFFICULT CONVERSATIONS

Given the differences between guidance for assessing symptomatic and asymptomatic patients and changes to guidance over time, it is little wonder patients and GPs find conversations about PSA testing challenging. GPs may be unclear about which guidance to follow for certain patients where symptoms are perhaps relatively mild. They are also mindful of the limitations of PSA in terms of the risks of both false positive and false negative results and the adverse consequences, and do not want to contribute to the problem of overdiagnosis of clinically insignificant prostate cancer. Some patients may interpret this hesitancy around PSA testing as GPs trying to dissuade or discourage them from having the test. UK GPs in particular are more hesitant around the use of PSA compared to international GP colleagues.^13^

Aside from age, the risk of prostate cancer is increased by a family history of the disease or being from Black African or Afro-Caribbean ancestry. Neither of these risk factors are addressed by NG12 or PCRMP guidance at all, leaving GPs in the dark about whether to change their approach for these patients and how. Making decisions about investigating patients in their late 70s and older is another grey area where GPs need to rely on clinical judgement and knowledge of the patient’s general health and preferences for potentially invasive diagnostic testing and treatments, should a diagnosis of prostate cancer be made. If the new NICE PSA thresholds were adopted for asymptomatic males as well, a significant proportion of males with clinically significant prostate cancer would be missed.^14^

## SEEKING CLARITY

GPs need clear, consistent national guidance to identify males at higher risk of clinically significant prostate cancer who would benefit from a 2-week wait referral and to reduce variation in practice across the NHS. Prostate Cancer UK have developed a package of educational materials to help GPs navigate the differences in current clinical guidance for the detection of prostate cancer in primary care.^15^ The charity is also seeking to fund research to transform prostate cancer diagnosis and generate the evidence for a nationally commissioned prostate cancer screening programme. Implementation of prostate MRI and new prostate biopsy approaches are reducing the harms for males going through the diagnostic pathway, although the optimal prostate cancer diagnostic pathway design is still being refined.

Also, the use of active surveillance to manage 90% of low-risk prostate cancer means these males avoid the consequences of radical therapy in the UK.^16^ The generation of primary care evidence on the use of PSA and other tests for early detection of prostate cancer could also help to refine clinical guidance for GPs and improve outcomes for patients.
